# O Impacto da Cardiopatia Grave nas Causas de Óbito e Sobrevida após Aposentadoria por Invalidez

**DOI:** 10.36660/abc.20240068

**Published:** 2024-09-17

**Authors:** Cesar Romaro Pozzobon, Gabriel Porto Soares, Ronir Raggio Luiz, Gláucia Maria Moraes de Oliveira

**Affiliations:** 1 Universidade Federal do Rio de Janeiro Rio de Janeiro RJ Brasil Universidade Federal do Rio de Janeiro, Rio de Janeiro, RJ – Brasil; 2 Universidade Federal do Rio de Janeiro Instituto do Coração Edson Abdala Saad Rio de Janeiro RJ Brasil Universidade Federal do Rio de Janeiro – Instituto do Coração Edson Abdala Saad, Rio de Janeiro, RJ – Brasil; 3 Universidade de Vassouras Vassouras RJ Brasil Universidade de Vassouras, Vassouras, RJ – Brasil

**Keywords:** Mortalidade, Aposentadoria, Cardiopatias, Sobrevida

## Abstract

**Fundamento:**

As doenças não comunicáveis são responsáveis por mortes prematuras e limitações. A aposentadoria por invalidez está associada a condições crônicas, especialmente a doenças cardiovasculares. A II Diretriz Brasileira de Cardiopatia Grave definiu critérios para enquadramento das doenças cardiovasculares. Poucos estudos abordam esse tema em instituições federais.

**Objetivos:**

Avaliar sobrevida e causas de óbito de servidores aposentados por invalidez na UFRJ, com ênfase no impacto da cardiopatia grave.

**Métodos:**

Estudo de coorte retrospectivo baseado nos registros de aposentadorias e óbitos ao longo de 15 anos. As aposentadorias foram divididas em três grupos: integral por cardiopatia grave, integral por outras doenças e proporcional. As causas de óbito foram obtidas a partir das certidões de óbito. Foram avaliadas taxa de mortalidade, sobrevida e a presença de diagnósticos concordantes entre a aposentadoria e o óbito. Foram utilizados testes qui-quadrado, log-rank, modelos de Cox e curvas de Kaplan-Meier. Significância estatística com intervalo de confiança de 95%, considerando p < 0,05.

**Resultados:**

Foram 630 aposentadorias, 368 (51,4%) no sexo feminino, com idade média de idade de 52,9 (DP=7,8) anos, e 169 (26,8%) óbitos. A mortalidade foi maior nos professores (37,0%; p=0,113), na faixa etária entre 65 e 70 anos (48,4%; p=0,004), no sexo masculino (34,0%; p=0,001), e nas aposentadorias integrais por cardiopatia grave (41,5%; p < 0,001). Diagnósticos concordantes entre aposentadoria e óbito foram mais frequentes em professores (74,1%; p=0,026) e nas aposentadorias integrais por cardiopatia grave (72,7%; p < 0,001).

**Conclusões:**

O diagnóstico de cardiopatia grave confere maior taxa de mortalidade e menor sobrevida aos aposentados por invalidez, e sua presença em maior frequência nos diagnósticos de aposentadoria e óbito ressalta sua importância neste contexto.

## Graphical abstract

**Figure:**
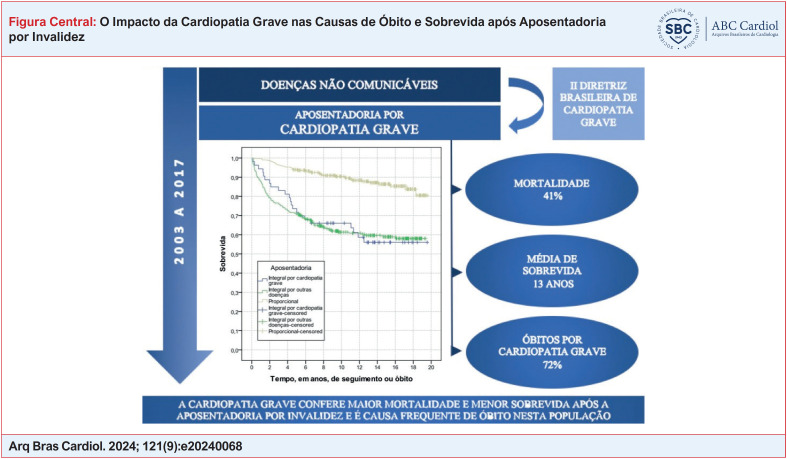

## Introdução

As doenças não comunicáveis (DNC) são um grave problema global, causando mortes prematuras, perda de qualidade de vida e impactos econômicos significativos.^1-3^ A Organização Mundial da Saúde destaca sua prevalência, sendo a principal causa de mortalidade, incapacidade prematura e aposentadoria por invalidez em muitos países, incluindo o Brasil.^4-6^

Responsáveis por aproximadamente 70% das mortes globais, as DNC incluem doenças cardiovasculares, neoplasias, doenças respiratórias crônicas e diabetes. As cardiovasculares lideram as estatísticas, representando 45% das mortes em 2019, segundo a "Estatística Cardiovascular 2021".^5^ Essa tendência é observada de maneira semelhante no Brasil, onde 72% das mortes são atribuídas as DNC, sendo 30% relacionadas a doenças cardiovasculares e 16% a neoplasias.^7^

A aposentadoria por incapacidade permanente ou invalidez representa um benefício destinado aos servidores públicos civis no Brasil, cuja inaptidão laboral de caráter duradouro é atribuída a doença ou acidente.^8^ A Lei nº 8.112 estabelece que a concessão de proventos integrais para tal aposentadoria se aplica nos casos derivados de acidente em serviço, moléstia profissional ou doença grave previamente elencada em legislação específica. Concomitantemente, proventos proporcionais são contemplados nas demais circunstâncias.^8^

A reforma previdenciária, implementada por meio da Emenda Constitucional nº 103,^9^ introduziu alterações significativas, restringindo o direito à aposentadoria integral apenas aos casos de incapacidade permanente decorrente de acidente de trabalho, doença profissional e doença do trabalho, gerando preocupações sobre a redução de rendimentos para os aposentados por invalidez.^10^

Diversas pesquisas internacionais identificaram uma maior mortalidade entre os aposentados por invalidez em comparação com aqueles que não se aposentaram, embora a razão para essa discrepância não esteja completamente esclarecida. Como justificativa, esses estudos levantaram a hipótese de que o fator determinante para essa diferença poderia ser a condição subjacente que levou à aposentadoria por invalidez. Contudo, não conseguiram estabelecer essa relação de maneira independente de outros fatores de confusão.^11-16^

Em 2006, com o objetivo de padronizar a interpretação e o enquadramento legal das doenças cardiovasculares, a Sociedade Brasileira de Cardiologia (SBC) desenvolveu a II Diretriz Brasileira de Cardiopatia Grave. Esta diretriz estabelece uma definição e categorização das cardiopatias com base em sua gravidade.^17^ O conceito de cardiopatia grave engloba condições cardíacas tanto crônicas quanto agudas, considerando as limitações na capacidade física e funcional impostas pela doença. Dentro desse contexto, e considerando que as doenças cardiovasculares são as principais DNC associadas à aposentadoria por invalidez e à mortalidade no Brasil, a definição de cardiopatia grave possibilita a avaliação da mortalidade específica entre os servidores aposentados por invalidez. Isso permite comparações com outras causas de mortalidade dentro desse mesmo grupo populacional.

O estudo foca na Universidade Federal do Rio de Janeiro (UFRJ), uma das principais universidades federais no Brasil,^18^ explorando a sobrevida e causas de óbito de servidores aposentados por invalidez de 2003 a 2017, com ênfase na influência da cardiopatia grave. Essa pesquisa visa preencher lacunas nacionais, especialmente entre servidores universitários.

## Métodos

Realizamos um estudo de coorte retrospectiva com base nas aposentadorias por invalidez de servidores públicos civis da UFRJ, entre janeiro de 2003 e dezembro de 2017, seguindo a metodologia de Pozzobon et al.^19^

As informações sobre óbitos foram obtidas do Subsistema Integrado de Atenção à Saúde do Servidor, SIAPENET, Sistema Integrado de Recursos Humanos, Certidões de Óbito da Coordenação de Gestão de Pessoal da UFRJ e Sistema de Informações sobre Mortalidade – SIM^20^ do Estado do Rio de Janeiro.

Enfatizando o impacto da cardiopatia grave, dividimos as aposentadorias em três grupos: integral por cardiopatia grave, integral por outras doenças e proporcional. Especificamente no grupo de aposentadorias integrais por cardiopatia grave, analisamos o diagnóstico de doença isquêmica do coração (DIC) como causa da aposentadoria e óbito. Consideramos a causa básica e associada para óbitos por cardiopatia grave. Para analisar a taxa de mortalidade nas aposentadorias integrais, seguimos a metodologia de Pozzobon et al.^19^ Os períodos de ocorrência das aposentadorias foram divididos e considerados a partir da data de publicação da II Diretriz Brasileira de Cardiopatia Grave, em agosto de 2006.

### Análise estatística

Utilizamos os softwares Excel-Microsoft® versão 16 para coleta de dados e Statistical Package for Social Sciences - SPSS® versão 24 para análises estatísticas. Resultados foram expressos em números absolutos, percentuais e mediana. O teste qui-quadrado comparou variáveis categóricas. Pelo teste Kolgomorov-Smirnov a distribuição da população não foi normal, com p < 0,001. Taxas de mortalidade foram calculadas considerando o tempo total até o óbito. O tempo médio de seguimento foi 10 anos e 6 meses. A sobrevida foi descrita com curvas de Kaplan-Meier e comparada pelo teste log-rank. Modelos de Cox univariado e múltiplo ajustaram a análise de mortalidade, com cálculo de hazard ratio (HR) bruto e ajustado (HRaj). Teste qui-quadrado avaliou a presença de diagnósticos concordantes na aposentadoria e óbito. Consideramos significativo p < 0,05 com intervalo de confiança de 95%.

## Resultados

Foram examinadas 700 aposentadorias por invalidez ocorridas entre janeiro de 2003 e dezembro de 2017. Em 70 registros (10%), os servidores foram revertidos à condição de ativos, sendo assim excluídos da análise, resultando em 630 aposentadorias consideradas. Desse total, 334 (53%) foram classificadas como aposentadorias integrais e 296 (47%) como proporcionais.

Ao analisar os dados, independentemente do tipo de aposentadoria, constatou-se que 499 servidores (79,2%) estavam na faixa etária de 30 a 59 anos no momento da aposentadoria, 77 (12,2%) estavam na faixa de 60 a 64 anos, e 54 (8,6%) estavam na faixa de 65 a 70 anos. No que se refere ao gênero, 262 (41,6%) ocorreram no sexo masculino e 368 (51,4%) no feminino. A mediana de idade na aposentadoria foi de 53 (47-58) anos.

Foram identificados 169 óbitos, correspondendo a uma proporção de 26,8% dentro da amostra estudada. Ao considerar o cargo de entrada na universidade, a proporção de óbitos mostrou-se mais elevada entre os professores (37,0%) em comparação com os demais cargos. Além disso, a proporção de óbitos foi significativamente superior na faixa etária de 65 a 70 anos (48,4%) em relação às outras faixas, apesar de o maior número absoluto de aposentadorias ter ocorrido em idades mais jovens. Da mesma forma, a proporção dos óbitos também foi maior no sexo masculino (34,0%), assim como nas aposentadorias integrais devido a cardiopatia grave (41,5%), em comparação com as proporcionais. Não houve diferença na mortalidade em relação ao período da aposentadoria. Estes achados podem ser observados na Tabela 1 a seguir.

**Tabela 1:** Mortalidade da coorte de ex-servidores da UFRJ aposentados por invalidez de 2003 a 2017 segundo o cargo, a idade, o sexo e o perfil das aposentadorias

Cargo, idade, sexo e o perfil das aposentadorias		Total		Óbito*				p-valor pelo teste χ^2^
				Não		Sim		
		(n=630; 100%)		(n=461; 73,2%)		(n=169; 26,8%)		
		N	%	n	%	N	%	
**Cargo**								
	Professor	73	100,0	46	63,0	27	**37,0**	0,113
	Tec-adm superior	92	100,0	69	75,0	23	**25,0**	
	Tec-adm médio ou elementar	465	100,0	346	74,4	119	**25,6**	
**Idade na aposentadoria**								
	30 a 59 anos	499	100,0	380	76,2	119	**23,8**	0,004
	60 a 64 anos	77	100,0	48	62,3	29	**37,7**	
	65 a 70 anos	64	100,0	33	51,6	31	**48,4**	
**Sexo**								
	Masculino	262	100,0	173	66,0	89	**34,0**	0,001
	Feminino	368	100,0	288	78,3	80	**21,7**	
**Tipo de aposentadoria**								
	Integral por cardiopatia grave	53	100,0	31	58,5	22	**41,5**	< 0,001
	Integral por outras doenças	281	100,0	171	60,9	110	**39,1**	
	Proporcional	296	100,0	259	87,5	37	**12,5**	
**Período da aposentadoria**†								
	Até agosto de 2006	205	100,0	147	71,7	58	**28,3**	0,564
	A partir de setembro de 2006	425	100,0	314	73,9	111	**26,1**	

Tec-adm: Técnico-administrativos.

* Informação atualizada até julho de 2022.

† Em função da II Diretriz Brasileira de Cardiopatia Grave.

O cálculo das taxas de mortalidade corroborou os achados anteriores e podem ser encontrados na Tabela 2. Foram maiores no cargo de professor, na faixa etária entre 65 a 70 anos, no sexo masculino e nas aposentadorias integrais por cardiopatia grave. Em relação ao período de aposentadoria, a taxa de mortalidade foi maior nos servidores aposentados após agosto de 2006, quando houve mudança nos critérios de enquadramento das aposentadorias por cardiopatia grave, após a elaboração da II Diretriz Brasileira de Cardiopatia Grave.

**Tabela 2:** Taxas de mortalidade da coorte de ex-servidores da UFRJ aposentados por invalidez de 2003 a 2017 segundo o cargo, a idade na aposentadoria, o sexo e o perfil das aposentadorias

Cargo, idade na aposentadoria, sexo e tipo de aposentadoria		Óbitos*	Pessoas-ano	Taxa (IC95%) por 100 pessoas-ano		Média de Sobrevida (anos)	p-valor do teste log-rank
**Cargo**							
	Professor	27	695,3	**3,9**	(2,6 - 5,6)	13,4	0,094
	Tec-adm superior	23	948,7	**2,4**	(1,6 - 3,6)	15,3	
	Tec-adm médio ou elementar	119	4.995,0	**2,4**	(2,0 - 2,8)	15,4	
**Idade na aposentadoria**							
	30 a 59 anos	119	5.513,4	**2,2**	(1,8 - 2,6)	15,7	0,001
	60 a 64 anos	29	631,7	**4,6**	(3,1 - 6,5)	13,0	
	65 a 70 anos	31	493,9	**6,3**	(4,3 - 8,8)	13,0	
**Sexo**							
	Masculino	89	2.472,4	**3,6**	(2,9 - 4,4)	13,9	< 0,001
	Feminino	80	4.166,6	**1,9**	(1,5 - 2,4)	16,0	
**Tipo de aposentadoria**							
	Integral por cardiopatia grave	22	517,6	**4,3**	(2,7 - 6,3)	13,1	< 0,001
	Integral por outras doenças	110	2.575,1	**4,3**	(3,5 - 5,1)	12,8	
	Proporcional	37	3.546,3	**1,0**	(0,7 - 1,4)	17,7	
**Período da aposentadoria**†							
	Até agosto de 2006	58	2.934,2	**2,0**	(1,5 - 2,5)	15,5	0,364
	A partir de setembro de 2006	111	3.704,8	**3,0**	(2,5 - 3,6)	12,5	
**TOTAL**		**169**	**6.639,0**	**2,5**	**(2,2 - 2,9)**	**15,2**	

Tec-adm: Técnico-administrativos; IC: intervalo de confiança.

* Informação atualizada até julho de 2022.

† Em função da II Diretriz Brasileira de Cardiopatia Grave.

**Figura 1:**
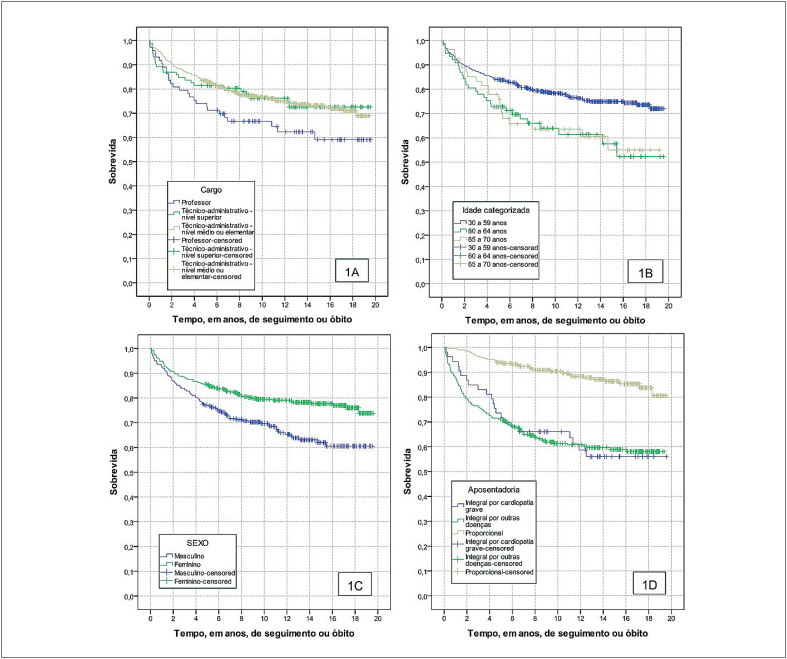
Curvas de sobrevivência da coorte de ex-servidores da UFRJ aposentados por invalidez no período de 2003 a 2017 segundo cargo (1A), idade na aposentadoria (1B), sexo (1C) e tipo de aposentadoria (1D).

As curvas de Kaplan-Meier na Figura 1 demonstram as diferenças observadas na média de sobrevida em relação as variáveis analisadas. Foram evidenciadas médias de sobrevida maiores nos cargos de técnico-administrativos de nível superior e nível médio ou elementar quando comparadas aos professores. Na faixa etária entre 30 e 59 anos a média de sobrevida foi de 15,7 anos e significativamente maior em relação as outras faixas etárias. Na comparação entre os sexos, a média de sobrevida foi maior no sexo feminino. Já de acordo com o tipo de aposentadoria, a média de sobrevida foi menor nas aposentadorias integrais por cardiopatia grave, em relação ao observado nas aposentadorias proporcionais.

Adicionalmente, para análise da mortalidade em função das variáveis, foram ajustados modelos de Cox univariado e múltiplo. Esta avaliação permitiu observar que para cada ano após a aposentadoria, a chance de óbito aumenta em 1,057 (5,7%) quando não ajustada, e em 1,045 (4,5%) quando ajustada com p < 0,001. Da mesma maneira, a chance de óbito é maior dentro do sexo masculino (HR_aj_ = 1,50) e quando a aposentadoria é integral por cardiopatia grave (HR_aj_ = 2,80) em comparação a aposentadoria proporcional (Tabela suplementar 1).

As taxas de mortalidade também foram calculadas na coorte de servidores aposentados de forma integral e comparadas para cada uma das variáveis. Nesta análise as taxas de mortalidade foram semelhantes entre os cargos de ingresso na universidade. Em relação a idade na ocasião da aposentadoria, as taxas de mortalidade foram maiores entre as faixas etárias de 60 a 64 anos e de 65 a 70 anos, assim como no sexo masculino. Dentro das aposentadorias integrais, as doenças foram divididas em grupos, com o grupo das neoplasias, hepatopatias ou nefropatias apresentando maior taxa de mortalidade, com as neoplasias sendo responsáveis por 87% dos óbitos dentro deste grupo, seguido do grupo das cardiopatias graves. Considerando o período de ocorrência da aposentadoria integral, a taxa de mortalidade observada após 2006 também foi maior (Tabela suplementar 2).

**Figura 2:**
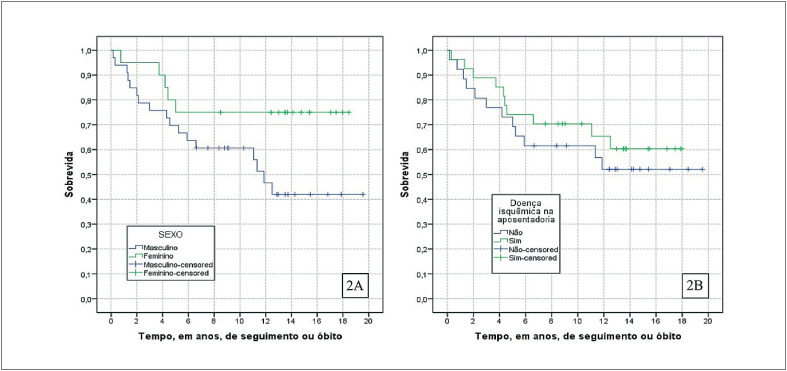
Curvas de sobrevida da coorte de ex-funcionários da UFRJ aposentados por cardiopatia grave no período de 2003 a 2017 segundo sexo (2A) e presença de cardiopatia isquêmica no momento da aposentadoria (2B).

As taxas de mortalidade foram avaliadas para o grupo de aposentadorias integrais por cardiopatia grave (n = 53) de acordo com a presença de DIC na ocasião da aposentadoria e para as variáveis sexo, idade e período de ocorrência da aposentadoria. Foram 22 óbitos, dos quais 10 em ex-servidores que apresentavam DIC no ato da aposentadoria conferindo menor taxa de mortalidade, maior média de sobrevida, entretanto sem diferença estatisticamente significativa. Em relação ao sexo foi observada maior taxa de mortalidade e menor média de sobrevida no sexo masculino (n = 17), conforme demonstrado na Figura 2. Não houve relevância significativa na análise das variáveis idade e período de aposentadoria, o que pode ser observado na Tabela 3.

**Tabela 3:** Taxas de mortalidade da coorte de ex-servidores da UFRJ aposentados por cardiopatia grave (n = 53) de 2003 a 2017 segundo a presença de doença isquêmica, idade na aposentadoria, sexo e período da aposentadoria

Doença isquêmica, idade na aposentadoria, sexo e período da aposentadoria		Óbitos*	Pessoas-ano	Taxa (IC95%) por 100 pessoas-ano		Média de sobrevida (anos)	p-valor pelo teste Log-rank
**Doença isquêmica**							
	Não	12	240,8	**5,0**	(2,7 - 8,5)	12,4	0,480
	Sim	10	276,9	**3,6**	(1,8 - 6,4)	13,0	
**Idade na aposentadoria**							
	30 a 59 anos	17	386,3	**4,4**	(2,6 - 6,9)	12,4	0,948
	60 a 64 anos	3	85,5	**3,5**	(0,9 - 9,5)	13,5	
	65 a 70 anos	2	45,8	**4,4**	(0,7 - 14,4)	10,2	
**Sexo**							
	Masculino	17	281,8	**6,0**	(3,6 - 9,5)	11,5	0,055
	Feminino	5	235,8	**2,1**	(0,8 - 4,7)	14,7	
**Período da aposentadoria**†							
	Até agosto de 2006	10	169,2	**5,9**	(3,0 - 10,5)	10,6	0,112
	A partir de setembro de 2006	12	348,4	**3,4**	(1,9 - 5,8)	11,8	
**TOTAL**		**22**	**517,6**	**4,3**	**(2,7 - 6,3)**	**13,2**	

* Informação atualizada até julho de 2022.

† Em função da II Diretriz Brasileira de Cardiopatia Grave.

**Figura 3:**
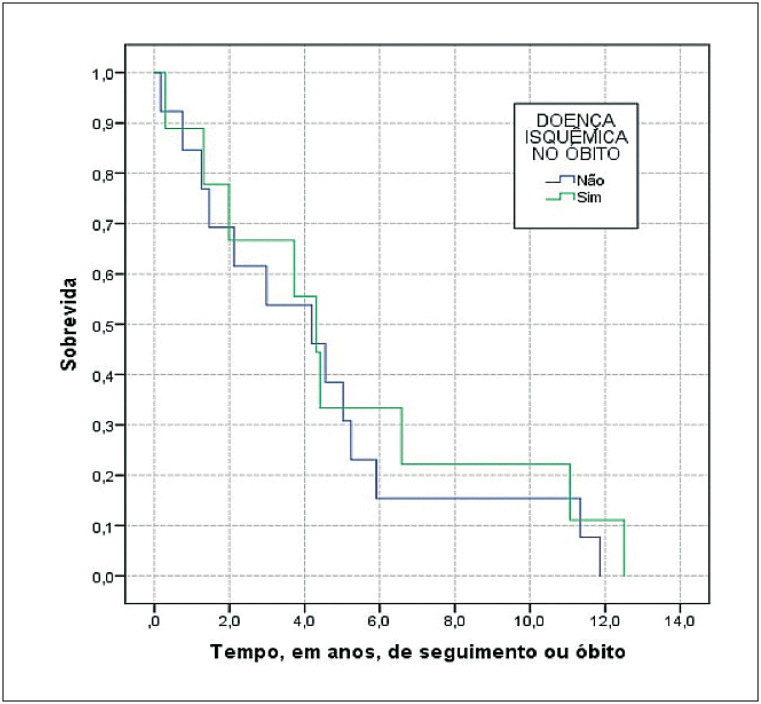
Curvas de sobrevida da coorte de ex-funcionários da UFRJ aposentados por doença cardíaca grave (n = 22) de 2003 a 2017 segundo óbito por doença isquêmica.

A análise das taxas de mortalidade também foi realizada entre os 22 óbitos que ocorreram no grupo de aposentadorias integrais por cardiopatia grave segundo a presença de DIC na ocasião do óbito conforme demonstrado no Tabela 4. Foram 9 óbitos por DIC, com menor taxa de mortalidade e maior média de sobrevida, porém sem significado estatístico, de acordo com a Figura 3. Em relação aos dados observados na tabela anterior, dois ex-servidores que apresentavam DIC na ocasião da aposentadoria faleceram de causas não cardiológicas, um por causa infecciosa e outro por neoplasia. Por outro lado, um ex-servidor aposentado por cardiopatia grave de etiologia não isquêmica, faleceu por infarto agudo do miocárdio configurando a presença de DIC na ocasião do óbito justificando os resultados.

**Tabela 4:** Taxas de mortalidade da coorte de ex-servidores da UFRJ falecidos por cardiopatia grave (n = 22) de 2003 a 2017 segundo o óbito por doença isquêmica

Óbito por doença isquêmica		Óbitos*	Pessoas-ano	Taxa (IC95%) por 100 pessoas-ano		Média de sobrevida (anos)	p-valor pelo teste Log-rank
	**Não (n=13)**	13	56,8	22,9	(12,7 - 38,2)	4,4	0,631
	**Sim (n=9)**	9	46,2	19,5	(9,5 - 35,9)	5,1	
**TOTAL (n=22)**		**22**	**103,0**	**21,4**	**(13,7 - 31,8)**	**4,7**	

* Informação atualizada até julho de 2022.

Por último, a presença de diagnósticos concordantes na aposentadoria e no óbito foi avaliada e comparada de acordo com cada variável. Houve diagnósticos concordantes em mais de 70% das aposentadorias no cargo de professor em comparação aos demais. Em contrapartida, os servidores de nível médio ou elementar, em sua maioria, não morreram da mesma doença pela qual se aposentaram. Em relação ao tipo de aposentadoria, houve diagnósticos concordantes em 72,7%, nas aposentadorias integrais por cardiopatia grave. Não houve diferença estatisticamente significativa na comparação entre as variáveis idade, sexo e período de ocorrência da aposentadoria. A Tabela 5 a seguir demonstra os dados obtidos.

**Tabela 5:** Ex-servidores da UFRJ aposentados por invalidez de 2003 a 2017 e já falecidos*, por diagnósticos concordantes de aposentadoria e óbito, segundo o cargo, a idade, o sexo e o perfil das aposentadorias

Cargo, idade na aposentadoria, sexo, tipo e período da aposentadoria		Total		Diagnósticos concordantes de aposentadoria e óbito?				p-valor pelo teste χ^2^
				Não		Sim		
		N	%	N	%	n	%	
**Cargo**								
	Professor	27	100,0	7	25,9	20	74,1	0,026
	Tec-adm superior	23	100,0	7	30,4	16	69,6	
	Tec-adm médio ou elementar	119	100,0	60	50,4	59	49,6	
**Idade na aposentadoria**								
	30 a 59 anos	119	100,0	52	43,7	67	56,3	0,596
	60 a 64 anos	29	100,0	11	37,9	18	62,1	
	65 a 70 anos	21	100,0	11	52,4	10	47,6	
**Sexo**								
	Masculino	89	100,0	43	48,3	46	51,7	0,211
	Feminino	80	100,0	31	38,8	49	61,3	
**Tipo de aposentadoria**								
	Integral por cardiopatia grave	22	100,0	6	45,5	16	72,7	< 0,001
	Integral por outras doenças	110	100,0	33	30,0	77	70,0	
	Proporcional	37	100,0	31	83,8	6	16,2	
**Período da aposentadoria**†								
	Até agosto de 2006	58	100,0	30	51,7	28	48,3	0,133
	A partir de setembro de 2006	111	100,0	44	39,6	67	60,4	
**TOTAL**		**169**	**100,0**	**74**	**43,8**	**95**	**56,2**	

Tec-adm: Técnico-administrativos.

* Informação atualizada até julho de 2022.

† Em função da II Diretriz Brasileira de Cardiopatia Grave.

## Discussão

Este estudo retrospectivo de coorte investigou a sobrevida e mortalidade de servidores aposentados por invalidez na UFRJ de 2003 a 2017, com foco na influência da cardiopatia grave. A inclusão da categorização das cardiopatias graves pela SBC proporcionou um instrumento valioso para analisar as condições cardiovasculares no contexto da aposentadoria por invalidez e mortalidade, fornecendo uma base essencial para análises específicas. Poucos trabalhos no âmbito nacional estudaram a mortalidade entre servidores aposentados, e os que se propuseram a fazer avaliaram servidores regidos pelo Regime Geral da Previdência Social, diferentemente do presente estudo, que avaliou servidores aposentados pelo Regime Jurídico Único (RJU).^10,21^ Outros estudos internacionais também analisaram a mortalidade entre aposentados por invalidez, entretanto esta comparação é difícil, uma vez que as leis que regem o sistema de aposentadoria e determinam invalidez são diferentes, assim como as características das populações estudadas.^11,22,23^ Além disso, o diferencial deste estudo foi analisar, dentre os servidores falecidos, a relação entre a doença que motivou a aposentadoria e a doença que determinou o óbito do servidor, com base nos dados contidos na certidão de óbito de cada servidor, garantindo assim a veracidade da informação, e consequentemente dos resultados.

Apesar de não ser o objetivo principal deste trabalho, é importante relatar que dentre as 630 aposentadorias analisadas, independentemente do tipo, a maior ocorrência da invalidez se deu na faixa etária mais precoce, de 30 a 59 anos. Estes achados já foram descritos em outros estudos e reforçam o fato de que as DNC estão sendo responsáveis pela retirada precoce dos servidores do mercado de trabalho.^24,25^ Neste estudo, a invalidez também ocorreu mais no sexo feminino e estes achados também foram relatados em um trabalho realizado na Universidade Estadual de Londrina em 2016,^26^ assim como em outros estudos nacionais e internacionais.^27,28^ Presume-se que essa discrepância derive do fato das mulheres, ao longo de suas vidas profissionais, desempenharem não apenas atividades laborais, mas também tarefas domésticas, o que amplia a carga de trabalho e os problemas de saúde associados.

Na avaliação da proporção de óbitos e da taxa de mortalidade, assim como da média de sobrevida dos servidores após a aposentadoria, os homens apresentaram tanto mortalidade, quanto probabilidade de morte maiores em relação as mulheres, com menor média de sobrevida, o que pode indicar que os homens são aposentados em condições de saúde mais precárias do que as mulheres.^29^ No entanto, generalizações sobre comportamentos e atitudes com base no gênero podem ser simplistas e não levar em conta a diversidade individual. Além disso, as características mencionadas podem ser influenciadas por uma variedade de fatores, como contexto cultural, social, econômico e educacional. Portanto, é fundamental abordar tais afirmações com cautela e reconhecer a complexidade das variáveis envolvidas.

No presente estudo uma maior taxa de mortalidade e uma menor sobrevida foram encontradas no cargo de professor e na faixa etária entre 65 e 70 anos. No que diz respeito aos professores, a legislação confere a eles o direito de dedicar dois terços de sua carga horária ao ensino em sala de aula, enquanto o terço restante é destinado à produção científica. Além disso, têm o direito a 45 dias de férias por ano, ao contrário dos demais servidores. Ao longo de suas carreiras, essa prerrogativa pode resultar em um menor desgaste físico e mental para essa categoria.^30,31^ Assim, professores teriam uma vida funcional ativa mais longa, e desta forma se afastariam do trabalho apenas quando acometidos por doenças mais graves, responsáveis pela invalidez, e desta forma com maior probabilidade de morte após a aposentadoria. Já no que diz respeito a faixa etária, era de se esperar uma maior mortalidade em idades mais avançadas conforme observado neste trabalho.

A proporção de óbitos, assim como a taxa de mortalidade foram maiores no grupo de aposentadorias integrais quando comparadas ao grupo de proporcionais, tanto no grupo das aposentadorias integrais por cardiopatia grave quanto no grupo das integrais por outras doenças. Na comparação por grupos de doenças entre as aposentadorias integrais, o grupo das neoplasias apresentou maior taxa de mortalidade, seguido da cardiopatia grave. Estes achados são concordantes com o observado na literatura, pois as doenças cardiovasculares ainda representam a principal causa de mortalidade em países desenvolvidos e em desenvolvimento, com uma tendência de redução na incidência e na mortalidade, ao passo que as neoplasias, como causa de óbito, vêm crescendo em todo mundo e já representam a segunda causa de morte na maioria dos países. Em países desenvolvidos e com população mais velha, projeta-se que em breve, as neoplasias ultrapassarão as doenças cardiovasculares.^32^

Além das análises abordadas anteriormente, cabe destacar os achados relacionados à DIC no contexto das aposentadorias por cardiopatia grave. Entre os servidores aposentados por essa condição, a presença de DIC no momento da aposentadoria emergiu como um fator associado a taxas de mortalidade mais baixas e uma maior média de sobrevida destacando a importância da detecção precoce da DIC no cenário da aposentadoria, possivelmente impactando positivamente a trajetória pós-aposentadoria. Ao considerar a presença da DIC no momento do óbito, observou-se que servidores falecidos com essa condição apresentaram uma menor taxa de mortalidade e uma maior média de sobrevida, embora sem significância estatística.

Além dos custos para os governos, existem custos para indivíduos que se aposentaram devido à DIC. A redução da renda como resultado da aposentadoria por DIC provavelmente reduzirá o padrão de vida dos indivíduos e poderá acarretar maior risco de condições inadequadas de poupança para a aposentadoria. Estudo realizado na Austrália reportou que os trabalhadores que se aposentaram precocemente devido a DIC tinham significativamente menos riqueza pessoal e poupanças mais baixas quando comparados com aqueles que permaneceram no mercado de trabalho até a aposentadoria.^33^ Esses achados nos fazem refletir sobre a necessidade de mudança das regras atuais de aposentadoria por cardiopatia grave devido a DIC.^17^

Ademais, a análise diferenciada por sexo revelou que os homens apresentaram uma maior taxa de mortalidade e uma menor média de sobrevida, indicando disparidades de risco entre os gêneros no grupo de aposentadorias por cardiopatia grave. Esses resultados ressaltam as complexas interações entre a doença cardíaca, aposentadoria e mortalidade, contribuindo para uma visão mais abrangente dos fatores que influenciam o desfecho desses servidores no pós-aposentadoria.

Por fim, na análise dos diagnósticos concordantes entre a doença que motivou a aposentadoria e causa do óbito do servidor, no cargo de professor houve coincidência entre os diagnósticos em mais de 70% dos casos, e isto pode ser justificado pelo fato dos professores se aposentarem por doenças mais graves e consequentemente com maior probabilidade de morte pela mesma doença. A congruência dentro do grupo de aposentadorias integrais também ocorreu em mais de 70% dos casos, principalmente no grupo integral por cardiopatia grave, denotando uma maior gravidade para as doenças que determinam este tipo de aposentadoria. Diferentemente deste trabalho, um estudo realizado na Suécia comparou a mortalidade de 1683 aposentados com a população não aposentada, ao longo de 18 anos. Uma taxa de mortalidade maior foi encontrada na população de aposentados por invalidez, entretanto esta diferença não pode ser atribuída a doença de base que ensejou a aposentadoria. Não houve associação clara entre o diagnóstico que motivou a incapacidade e a causa do óbito, sugerindo que a doença de base pode não ter relação com o desfecho desfavorável.^11^

A ausência de um grupo controle formado por servidores que se aposentaram voluntariamente por tempo de serviço constituiu uma limitação deste estudo para comparação de algumas variáveis. A escassez de outros estudos semelhantes com servidores regidos pelo RJU, ao mesmo tempo que torna o presente estudo original, acaba por limitar possíveis comparações de resultados representando também uma limitação. Este estudo pretende levantar hipóteses sobre aposentadorias por invalidez em idade produtiva e suas conclusões, embora limitadas aos dados da maior Universidade Federal do Brasil, podem auxiliar no desenvolvimento de novos estudos sobre o tema. Com as mudanças legislativas recentes, é essencial direcionar futuras pesquisas para compreender as implicações dessas alterações nas condições de vida dos servidores aposentados por invalidez.

## Conclusão

Este estudo proporcionou uma compreensão mais clara do padrão de mortalidade e sobrevida de servidores aposentados por invalidez na UFRJ. Destacou-se o impacto da cardiopatia grave, evidenciado nas proporções de óbitos e taxas de mortalidade, especialmente em aposentadorias integrais por essa condição. A presença acentuada de diagnósticos concordantes entre a causa da aposentadoria e a causa do óbito enfatiza a vulnerabilidade associada à cardiopatia grave. O risco de morte foi maior em aposentadorias integrais por cardiopatia grave. Por outro lado, a sobrevida foi mais prolongada em aposentadorias proporcionais e em faixas etárias mais precoces, sublinhando a importância de avaliações periódicas para possível reversão da aposentadoria, especialmente quando a incapacidade inicial se torna insubsistente (Figura Central).

## *Material suplementar

Para informação adicional, por favor, clique aqui.
