# Supplementary material for: O Impacto da Cardiopatia Grave nas Causas de Óbito e Sobrevida após Aposentadoria por Invalidez
Source: Arq Bras Cardiol. 2024 Sep 17;121(9):e20240068. [Article in Portuguese] doi: 10.36660/abc.20240068 (PMC11495571; doi:10.36660/abc.20240068)
Supplement: Supplementary file 1 [file 0066-782X-abc-121-09-e20240068-Suppl01.pdf]

Tabela suplementar 1. Modelos de Cox para a sobrevida em ex-servidores da UFRJ aposentados por invalidez de 2003 a 2017 segundo o cargo, a idade, o sexo e o perfil das aposentadorias.

**Cargo, idade, sexo e perfil das aposentadorias**	**Modelos de Cox univariados**		**Modelo de Cox múltiplo**	
	**HR**	**p-valor**	**HR_{aj}**	**p-valor**
**Idade na aposentadoria (anos)**	1,057	< 0,001	1,045	< 0,001
**Cargo**		0,098		0,518
Professor	1,58	0,033	0,79	0,318
Tec-adm superior	1,01	0,952	0,84	0,455
Tec-adm médio ou elementar	1		1	
**Sexo**				
Masculino	1,73	< 0,001	1,50	0,011
Feminino	1		1	
**Tipo de aposentadoria**		< 0,001		< 0,001
Integral por cardiopatia grave	3,84	< 0,001	2,80	< 0,001
Integral por outras doenças	3,89	< 0,001	3,59	< 0,001
Proporcional	1		1	
**Período da aposentadoria***				
Até agosto de 2006	0,86	0,365	0,91	0,570
A partir de setembro de 2006	1		1	

Tec-adm=Técnico-administrativos. HR= ***Hazard Ratio.*** **HR_{aj}=** ***Hazard Ratio*** **ajustado. *Em função da II Diretriz Brasileira de Cardiopatia Grave.**

Tabela suplementar 2. Taxas de mortalidade da coorte de ex-servidores da UFRJ com aposentadoria integral por invalidez de 2003 a 2017 segundo o cargo, a idade, o sexo e o perfil das aposentadorias.

**Cargo, idade na aposentadoria, sexo e tipo de aposentadoria**	**Óbitos***	**Pessoas-ano**	**Taxa (IC95%) por 100 pessoas-ano**		**Média de Sobrevida (anos)**
**Cargo**					
Professor	25	567,1	**4,4**	(2,9 - 6,4)	12,6
Tec-adm superior	21	488,5	**4,3**	(2,7 - 6,5)	13,0
Tec-adm médio ou elementar	86	2.037,1	**4,2**	(3,4 - 5,2)	12,9
**Idade**					
30 a 59 anos	95	2.466,1	**3,9**	(3,1 - 4,7)	13,1
60 a 64 anos	20	294,9	**6,8**	(4,2 - 10,3)	11,6
65 a 70 anos	17	331,8	**5,1**	(3,1 - 8,0)	11,4
**Sexo**					
Masculino	71	1.344,5	**5,3**	(4,1 - 6,6)	12,0
Feminino	61	1.748,2	**3,5**	(2,7 - 4,4)	13,4
**Grupos de doença**					
Cardiopatias graves	22	517,6	**4,3**	(2,7 - 6,3)	13,2
Neoplasias, hepatopatias ou nefropatias	71	1.001,1	**7,1**	(5,6 - 8,9)	10,3
Alienação mental	15	693,5	**2,2**	(1,2 - 3,5)	15,7
Incapacidades físicas	21	762,2	**2,8**	(1,7 - 4,1)	14,5
Hanseníase, SIDA ou tuberculose	3	118,3	**2,5**	(0,6 - 6,9)	14,2
**Período da aposentadoria**†					
Até agosto de 2006	45	1.481,2	**3,0**	(2,2 - 4,0)	13,7
A partir de setembro de 2006	87	1.611,5	**5,4**	(4,3 - 6,6)	10,4
**TOTAL**	**132**	**3.092,7**	4,3	**(3,6 - 5,0)**	**12,9**

Tec-adm=Técnico-administrativos. *Informação atualizada até julho de 2022. †Em função da II Diretriz Brasileira de Cardiopatia Grave.
