# Supplementary material for: O Impacto da Cardiopatia Grave nas Causas de Óbito e Sobrevida após Aposentadoria por Invalidez
Source: Arq Bras Cardiol. 2024 Sep 17;121(9):e20240068. [Article in Portuguese] doi: 10.36660/abc.20240068 (PMC11495571; doi:10.36660/abc.20240068)
Supplement: Supplementary file 2 [file 0066-782X-abc-121-09-e20240068-Suppl02.pdf]

Supplementary Table 1. Cox models for survival in former UFRJ employees retired due to disability from 2003 to 2017 according to position, age at retirement, gender, retirement profile and retirement period.

Position, age at retirement, gender, retirement profile and retirement period	Univariate Cox Model		Multiple Cox Model	
	HR	p-value	HR_{aj}	p-value
**Age at retirement (years)**	1,057	< 0,001	1,045	< 0,001
**Position**		0,098		0,518
Professor	1,58	0,033	0,79	0,318
Upper-level admin tech staff	1,01	0,952	0,84	0,455
Mid/elementary-level admin tech staff	1		1	
**Gender**				
Male	1,73	< 0,001	1,50	0,011
Female	1		1	
**Type of retirement**		< 0,001		< 0,001
Full due to severe heart disease	3,84	< 0,001	2,80	< 0,001
Full due to other disease	3,89	< 0,001	3,59	< 0,001
Proportional	1		1	
**Retirement period***				
Until August 2006	0,86	0,365	0,91	0,570
From September 2006	1		1	

Admin tech=Administrative technical. HR= *Hazard Ratio.* HR_{aj}= *Hazard Ratio* adjusted. * In accordance with the II Brazilian Guideline on Severe Cardiopathy.

Supplementary Table 2. Mortality rates of the cohort of former UFRJ employees with full disability retirement from 2003 to 2017 according to position, age at retirement, gender, retirement profile and retirement period.

Position, age at retirement, gender, retirement profile and retirement period	Deaths*	Person-years	Rate (95% CI) per 100 person-years		Mean Survival (years)
**Position**					
Professor	25	567,1	**4,4**	(2,9 - 6,4)	12,6
Upper-level admin tech staff	21	488,5	**4,3**	(2,7 - 6,5)	13,0
Mid/elementary-level admin tech staff	86	2.037,1	**4,2**	(3,4 - 5,2)	12,9
**Age at retirement**					
30 to 59 years	95	2.466,1	**3,9**	(3,1 - 4,7)	13,1
60 to 64 years	20	294,9	**6,8**	(4,2 - 10,3)	11,6
65 to 70 years	17	331,8	**5,1**	(3,1 - 8,0)	11,4
**Gender**					
Male	71	1.344,5	**5,3**	(4,1 - 6,6)	12,0
Female	61	1.748,2	**3,5**	(2,7 - 4,4)	13,4
**Group of diseases**					
Severe heart diseases	22	517,6	**4,3**	(2,7 - 6,3)	13,2
Neoplasms, liver or kidney diseases	71	1.001,1	**7,1**	(5,6 - 8,9)	10,3
Mental disorder	15	693,5	**2,2**	(1,2 - 3,5)	15,7
Physical disabilities	21	762,2	**2,8**	(1,7 - 4,1)	14,5
Leprosy, AIDS or tuberculosis	3	118,3	**2,5**	(0,6 - 6,9)	14,2
**Retirement period***					
Until August 2006	45	1.481,2	**3,0**	(2,2 - 4,0)	13,7
From September 2006	87	1.611,5	**5,4**	(4,3 - 6,6)	10,4
**TOTAL**	**132**	**3.092,7**	**4,3**	**(3,6 - 5,0)**	**12,9**

Admin tech=Administrative technical. CI= Confidence interval. *Information updated until July 2022. †In accordance with the II Brazilian Guideline on Severe Cardiopathy.
