# Supplementary material for: O Impacto da Cardiopatia Grave nas Causas de Óbito e Sobrevida após Aposentadoria por Invalidez
Source: Arq Bras Cardiol. 2024 Sep 17;121(9):e20240068. [Article in Portuguese] doi: 10.36660/abc.20240068 (PMC11495571; doi:10.36660/abc.20240068)
Supplement: Translation

# Translation: The Impact of Severe Heart Disease on Causes of Death and Survival after Disability Retirement

**DOI:** 10.36660/abc.20240068i

**Authors:** Cesar Romaro Pozzobon, Gabriel Porto Soares, Ronir Raggio Luiz, Gláucia Maria Moraes de Oliveira

**Affiliations:** 1 Rio de Janeiro RJ Brazil Universidade Federal do Rio de Janeiro, Rio de Janeiro, RJ – Brazil; 2 Rio de Janeiro RJ Brazil Universidade Federal do Rio de Janeiro – Instituto do Coração Edson Abdala Saad, Rio de Janeiro, RJ – Brazil; 3 Vassouras RJ Brazil Universidade de Vassouras, Vassouras, RJ – Brazil

**Keywords:** Mortality, Retirement, Heart Diseases, Survival

## Abstract

**Background:**

Noncommunicable diseases contribute to premature deaths and limitations. Disability retirement is linked to chronic conditions, particularly cardiovascular diseases. The II Brazilian Guideline for Severe Heart Disease established criteria for cardiovascular disease classification. However, there is a lack of research in this topic within federal institutions.

**Objectives:**

Evaluate the survival and causes of death among disabled retirees at UFRJ, focusing on the impact of severe heart disease.

**Methods:**

A retrospective cohort study based on retirement and death records over 15 years. Retirements were categorized into three groups: full retirement due to severe heart disease, full retirement due to other diseases and proportional. Causes of death were obtained from death certificates. Mortality rates, survival and the presence of matching diagnoses between retirement and death were evaluated. Chi-square, log-rank, Cox models, Kaplan-Meier curves were utilized. Statistical significance with a 95% confidence interval, considering p<0.05.

**Results:**

There were 630 retirements, 368 (51.4%) in females, with an average age of 52.9 (SD=7.8) years, and 169 (26.8%) deaths. Mortality was higher in professors (37.0%; p=0.113), in the age group between 65 and 70 years (48.4%; p=0.004), in males (34.0%; p=0.001), and in full retirements due to severe heart disease (41.5%; p<0.001). Matching diagnoses between retirement and death were more frequent in professors (74.1%; p=0.026) and in full retirements due to severe heart disease (72.7%; p<0.001).

**Conclusions:**

Severe heart disease diagnosis is associated with higher mortality and shorter survival in disabled retirees. Its frequent occurrence in retirement and death diagnoses underscores its significance in this context.

## Introduction

Noncommunicable diseases (NCDs) pose a significant global problem, leading to premature deaths, reduced quality of life, and substantial economic impacts.^1-3^ The World Health Organization highlights them as the leading cause of mortality, premature disability, and disability retirement in many countries, including Brazil.^4-6^

NCDs are responsible for approximately 70% of global deaths, and include cardiovascular diseases, neoplasms, chronic respiratory diseases, and diabetes. Cardiovascular diseases rank first, accounting for 45% of deaths in 2019, according to "Cardiovascular Statistics 2021".^5^ A similar trend is observed in Brazil, where 72% of deaths are attributed to NCDs, 30% of which are related to cardiovascular diseases and 16% to neoplasms.^7^

Permanent incapacity or disability retirement is a benefit granted to Brazilian civil public servants whose long-term work incapacity is attributed to illness or accident.^8^ Law n^o^ 8.112 establishes that full benefits apply when retirement is due to service-related accidents, occupational disease, or specifically nominated severe illnesses. In all other circumstances, limited benefits are provided.^8^

The pension reform implemented by Constitutional Amendment n^o^ 103^9^ introduced significant changes, restricting full retirement benefits to cases of permanent disability due to work-related accidents, occupational disease, or work-related illness, and raising concerns about disability retirees’ reduced income.^10^

Many studies worldwide have identified higher mortality rates among disability retirees when compared to those who did not retire, and the reason for this discrepancy remains unclear. The hypothesis of these studies was that the underlying condition leading to disability retirement could be the determining factor for this difference. However, this association was not significant after adjusting for confounding factors.^11-16^

In 2006, in an effort to standardize the interpretation of the legal framework regarding, the Brazilian Society of Cardiology (SBC) developed the II Brazilian Guideline on Severe Heart Disease. This guideline establishes that heart diseases are to be defined and classified by severity.^17^ The concept of severe heart disease encompasses both chronic and acute cardiac disorders, considering the physical and functional limitations imposed by the illness. The definition of severe heart disease enables the evaluation of specific mortality among disability retirees and allows comparisons with other causes of mortality within this population group. Cardiovascular disease is the leading cause of disability retirement and mortality in Brazil when NCDs are considered.

This study analyzes survival and causes of death among disability retirees from the Federal University of Rio de Janeiro (UFRJ), one of the leading federal universities in Brazil,^18^ from 2003 to 2017, specifically exploring the influence of severe heart disease. This research aims to fill national research gaps, especially regarding university employees.

## Methods

We conducted a retrospective cohort study of UFRJ civil public servants who retired due to disability from January 2003 to December 2017, following the methodology of Pozzobon et al.^19^

Information on deaths was obtained from the Integrated Health Care Subsystem for Civil Servants (SIAPENET), the Integrated Human Resources System, Death Certificates from the UFRJ Personnel Management Coordination, and the Mortality Information System (SIM)^20^ of the State of Rio de Janeiro.

Emphasizing the impact of severe heart disease, we divided retirees into three groups: full retirement due to severe heart disease, full retirement due to other diseases, and proportional retirement. Specifically, within the group of full retirements due to severe heart disease, we analyzed the diagnosis of ischemic heart disease (IHD) as the cause of retirement and death. We considered both primary and associated causes of death due to severe heart disease. To analyze the mortality rate in full retirements, we followed the methodology of Pozzobon et al.^19^ The timing of retirement occurrences was classified as either before or after the publication of the II Brazilian Guideline on Severe Heart Disease (August 2006).

### Statistical analysis

We used Excel-Microsoft® version 16 for data collection and Statistical Package for Social Sciences – SPSS® version 24 for statistical analyses. Results were expressed in absolute numbers, percentages, and medians. The chi-square test was used to compare categorical variables. According to the Kolmogorov-Smirnov test, the population distribution was not normal, with p < 0.001. Mortality rates were calculated considering the total time until death. The mean follow-up time was 10 years and 6 months. Survival was described using Kaplan-Meier curves and the log-rank test was used for comparisons. Univariate and multivariate Cox models were used to adjust for variables in the mortality analysis, with calculation of crude and adjusted hazard ratios (HR and HRaj). The chi-square test was used to evaluate the concordance of diagnoses at retirement and death. A p-value < 0.05 with a 95% confidence interval was considered significant.

## Results

We examined a total of 700 disability retirements occurring between January 2003 and December 2017. Seventy employees (10%) returned to active duty and were excluded from the analysis, resulting in 630 retirements being studied. Among these, 334 (53%) were classified as full retirements and 296 (47%) as proportional retirements.

The full sample consisted of 499 employees (79.2%) aged between 30 and 59 at the time of retirement, 77 (12.2%) aged between 60 and 64, and 54 (8.6%) aged between 65 and 70. Regarding gender, 262 (41.6%) were male and 368 (58.4%) were female. The median age at retirement was 53 years (range 47-58).

There were 169 deaths, corresponding to 26.8% of the studied sample. Considering the job position at the university, the proportion of deaths was higher among professors (37.0%) compared to other positions. Additionally, the proportion of deaths was significantly higher in the 65 to 70 age group (48.4%) compared to other age groups, despite the higher absolute number of retirements at younger ages. Similarly, the proportion of deaths was higher among males (34.0%) and in full retirements due to severe heart disease (41.5%) compared to proportional retirements. The timing of retirement was not associated with mortality. These findings are shown in Table 1 below.

**Table 1:** Mortality of the cohort of former UFRJ employees retired due to disability from 2003 to 2017 according to position, age at retirement, gender, retirement profile and retirement period

Position, age at retirement, gender and retirement profile		Total		Death*				p-value from the χ^2^ test
				No		Yes		
		(n=630; 100%)		(n=461; 73.2%)		(n=169; 26.8%)		
		N	%	n	%	N	%	
**Position**								
	Professor	73	100.0	46	63.0	27	**37.0**	0.113
	Upper-level admin tech staff	92	100.0	69	75.0	23	**25.0**	
	Mid/elementary-level admin tech staff	465	100.0	346	74.4	119	**25.6**	
**Age at retirement**								
	30 to 59 years	499	100.0	380	76.2	119	**23.8**	0.004
	60 to 64 years	77	100.0	48	62.3	29	**37.7**	
	65 to 70 years	64	100.0	33	51.6	31	**48.4**	
**Gender**								
	Male	262	100.0	173	66.0	89	**34.0**	0,001
	Female	368	100.0	288	78.3	80	**21.7**	
**Type of retirement**								
	Full due to severe heart disease	53	100.0	31	58.5	22	**41.5**	< 0.001
	Full due to other disease	281	100.0	171	60.9	110	**39.1**	
	Proportional	296	100.0	259	87.5	37	**12.5**	
**Retirement period**†								
	Until August 2006	205	100.0	147	71.7	58	**28.3**	0.564
	From September 2006	425	100.0	314	73.9	111	**26.1**	

Admin tech: Administrative technical.

* Information updated until July 2022.

† In accordance with the II Brazilian Guideline on Severe Cardiopathy.

The calculation of mortality rates corroborated the previous findings, as shown in Table 2. Mortality rates were higher among professors, in the 65 to 70 age group, among males, and in full retirements due to severe heart disease. Regarding the retirement period, the mortality rate was higher among employees who retired after August 2006.

**Table 2:** Mortality rates of the cohort of former UFRJ employees retired due to disability from 2003 to 2017 according to position, age at retirement, gender, retirement profile and retirement period

Position, age at retirement, gender and retirement profile		Deaths*	Person-years	Rate (95% CI) per 100 person-years		Mean Survival (years)	p-value of the log-rank test
**Position**							
	Professor	27	695.3	**3.9**	(2.6 - 5.6)	13.4	0.094
	Upper-level admin tech staff	23	948.7	**2.4**	(1.6 - 3.6)	15.3	
	Mid/elementary-level admin tech staff	119	4.995.0	**2.4**	(2.0 - 2.8)	15.4	
**Age at retirement**							
	30 to 59 years	119	5.513.4	**2.2**	(1.8 - 2.6)	15.7	0.001
	60 to 64 years	29	631.7	**4.6**	(3.1 - 6.5)	13.0	
	65 to 70 years	31	493.9	**6.3**	(4.3 - 8.8)	13.0	
**Gender**							
	Male	89	2.472.4	**3.6**	(2.9 - 4.4)	13.9	< 0.001
	Female	80	4.166.6	**1.9**	(1.5 - 2.4)	16.0	
**Type of retirement**							
	Full due to severe heart disease	22	517.6	**4.3**	(2.7 - 6.3)	13.1	< 0.001
	Full due to other disease	110	2.575.1	**4.3**	(3.5 - 5.1)	12.8	
	Proportional	37	3.546.3	**1.0**	(0.7 - 1.4)	17.7	
**Retirement period**†							
	Until August 2006	58	2.934.2	**2.0**	(1.5 - 2.5)	15.5	0.364
	From September 2006	111	3.704.8	**3.0**	(2.5 - 3.6)	12.5	
**TOTAL**		**169**	**6.639.0**	**2.5**	**(2.2 - 2.9)**	**15.2**	

Admin tech: Administrative technical; CI: confidence interval.

* Information updated until July 2022.

† In accordance with the II Brazilian Guideline on Severe Cardiopathy.

The Kaplan-Meier curves in Figure 1 illustrate the observed differences in mean survival in relation to the analyzed variables. Higher mean survival rates were observed among upper-level and mid/elementary-level administrative technical staff compared to professors. In the 30 to 59 age group, the mean survival was 15.7 years, significantly higher than in the other age groups. When comparing genders, mean survival was higher in females. Regarding type of retirement, mean survival was lower in full retirements due to severe heart disease compared to proportional retirements.

**Figure 1:** Survival curves of the cohort of former UFRJ employees retired due to disability from 2003 to 2017 according to position (1A), age at retirement (1B), gender (1C), and type of retirement (1D).

Additionally, to analyze mortality in relation to variables, Cox univariate and multivariate models were adjusted. For each year after retirement, the risk of death increased by 1.057 (5.7%) when unadjusted, and by 1.045 (4.5%) when adjusted, with p < 0.001. The risk of death was higher in males (HRadj = 1.50) and in cases of full retirement due to severe heart disease (HRadj = 2.80) compared to proportional retirement (Supplementary Table 1).

Mortality rates were also calculated in the cohort of fully retired employees and compared across various variables. In this analysis, mortality rates were similar across all job entry positions. Regarding age at retirement, mortality rates were higher in the 60 to 64 and 65 to 70 age groups, as well as among males. Within full retirements, diseases were divided into groups, with the group of neoplasms, liver and kidney diseases showing the highest mortality rate. Neoplasms accounted for 87% of deaths within this group, followed by the group of severe heart diseases. Considering the timing of retirement, the mortality rate was higher among those who retired after August 2006 (Supplementary Table 2).

Mortality rates were evaluated in the group of full retirements due to severe heart disease (n = 53) according to the presence of ischemic heart disease (IHD) at the time of retirement and for the variables of gender, age, and retirement time. There were 22 deaths, and 10 deceased employees had IHD at retirement, showing a lower mortality rate and higher mean survival, but this difference was not statistically significant. In relation to gender, a higher mortality rate and lower mean survival were observed in males (n = 17), as shown in Figure 2. There was no significant difference in mortality rates when age and retirement time were analyzed, as shown in Table 3.

**Figure 2:** Survival curves of the cohort of former UFRJ employees retired due to severe heart disease from 2003 to 2017 according to gender (2A) and presence of ischemic heart disease at the time of retirement (2B).

**Table 3:** Mortality rates of the cohort of former UFRJ employees retired due to severe heart disease (n = 53) from 2003 to 2017 according to the presence of ischemic heart disease, age at retirement, gender and retirement period

Ischemic heart disease, age at retirement, gender and retirement period		Deaths*	Person-years	Rate (95% CI) per 100 person-years		Mean Survival (years)	p-value of the log-rank test
**Ischemic heart disease**							
	No	12	240.8	**5.0**	(2.7 - 8.5)	12.4	0.480
	Yes	10	276.9	**3.6**	(1.8 - 6.4)	13.0	
Age at retirement							
	30 to 59 years	17	386.3	**4.4**	(2.6 - 6.9)	12.4	0.948
	60 to 64 years	3	85.5	**3.5**	(0.9 - 9.5)	13.5	
	65 to 70 years	2	45.8	**4.4**	(0.7 - 14.4)	10.2	
**Gender**							
	Male	17	281.8	**6.0**	(3.6 - 9.5)	11.5	0.055
	Female	5	235.8	**2.1**	(0.8 - 4.7)	14.7	
**Retirement period**†							
	Until August 2006	10	169.2	**5.9**	(3.0 - 10.5)	10.6	0.112
	From September 2006	12	348.4	**3.4**	(1.9 - 5.8)	11.8	
**TOTAL**		**22**	**517.6**	**4.3**	**(2.7 - 6.3)**	**13.2**	

CI: Confidence interval.

* Information updated until July 2022.

† In accordance with the II Brazilian Guideline on Severe Cardiopathy. Brasileira de Cardiopatia Grave.

The analysis of mortality rates was also conducted among the 22 deaths in the group of full retirements due to severe heart disease according to the presence of IHD at the time of death, as shown in Table 4. There were 9 deaths due to IHD, with a lower mortality rate and higher mean survival, but without statistical significance, as shown in Figure 3. Regarding the data observed in the previous table, two former employees who had IHD at the time of retirement died from non-cardiological causes, one due to an infectious disorder and the other due to neoplasia. Conversely, one former employee retired due to severe heart disease of non-ischemic etiology died from acute myocardial infarction, indicating the presence of IHD at the time of death and justifying the results.

**Table 4:** Mortality rates of the cohort of former UFRJ employees deceased due to severe heart disease (n = 22) from 2003 to 2017 according to death from ischemic heart disease

Death from ischemic heart disease		Deaths*	Person-years	Rate (95% CI) per 100 person-years		Mean Survival (years)	p-value of the log-rank test
	**No (n=13)**	13	56.8	22.9	(12.7 - 38.2)	4.4	0.631
	**Yes (n=9)**	9	46.2	19.5	(9.5 - 35.9)	5.1	
**TOTAL (n=22)**		**22**	**103.0**	**21.4**	**(13.7 - 31.8)**	**4.7**	

CI: confidence interval.

* Information updated until July 2022.

**Figure 3:** Survival curves of the cohort of former UFRJ employees retired due to severe heart disease (n = 22) from 2003 to 2017 according to death from ischemic disease.

Finally, concordant diagnoses at the time of retirement and death were found in over 70% of the retirements among professors; most medium or elementary level staff did not die from the same disease from which they retired. There were concordant diagnoses in 72.7% of the employees that retired due to severe heart disease. Concordance was not affected by age, sex, and time of retirement (Table 5).

**Table 5:** Former UFRJ employees retired due to disability from 2003 to 2017 and deceased*, with concordant diagnoses for retirement and death, according to position, age at retirement, gender, retirement profile and retirement period

Position, age, gender, retirement profile and retirement period		Total		Concordant diagnoses for retirement and death?				p-value from the χ^2^ test
				No		Yes		
		N	%	N	%	n	%	
**Position**								
	Professor	27	100.0	7	25.9	20	74.1	0,026
	Upper-level admin tech staff	23	100.0	7	30.4	16	69.6	
	Mid/elementary-level admin tech staff	119	100.0	60	50.4	59	49.6	
**Age at retirement**								
	30 to 59 years	119	100.0	52	43.7	67	56.3	0.596
	60 to 64 years	29	100.0	11	37.9	18	62.1	
	65 to 70 years	21	100.0	11	52.4	10	47.6	
**Gender**								
	Male	89	100.0	43	48.3	46	51.7	0.211
	Female	80	100.0	31	38.8	49	61.3	
**Type of retirement**								
	Full due to severe heart disease	22	100.0	6	45.5	16	72.7	< 0.001
	Full due to other disease	110	100.0	33	30.0	77	70.0	
	Proportional	37	100.0	31	83.8	6	16.2	
**Retirement period**†								
	Until August 2006	58	100.0	30	51.7	28	48.3	0.133
	From September 2006	111	100.0	44	39.6	67	60.4	
**TOTAL**		**169**	**100.0**	**74**	**43.8**	**95**	**56.2**	

Admin tech: administrative technicaxl.

* Information updated until July 2022.

† In accordance with the II Brazilian Guideline on Severe Cardiopathy.

## Discussion

This retrospective cohort study investigated the survival and mortality of employees retired due to disability at UFRJ from 2003 to 2017, focusing on the influence of severe heart disease. Proper definition of severe heart disease by SBC proved valuable for analyzing cardiovascular conditions in the context of disability retirement and mortality. Few brazilian studies have examined mortality among retired employees. Those that have typically evaluated employees under the General Social Security System, whereas here we studied employees retired under another specific work legislation, the Single Legal Regime (RJU).^10,21^ Additionally, other international studies have analyzed mortality among retirees due to disability. However, such comparisons are challenging due to differences in the laws governing retirement systems, definitions of disability, and the characteristics of studied populations.^11,22,23^ Moreover, the original contribution of this study was to analyze the relationship between the illness that led to retirement and the illness that cause the employee's death among deceased employees. This analysis was based on data from each employee's death certificate, ensuring the accuracy of the information and, consequently, the results.

Although not the main objective of this study, it is noteworthy that most of the 630 retirees analyzed, regardless of retirement type, were in the early age range (30 to 59 years). These findings are consistent with other studies, reinforcing that NCDs contribute to early workforce withdrawal.^24,25^ In this study, disability was also more frequent among females, a finding reported in a study at the State University of Londrina in 2016,^26^ as well as in other national and international studies.^27,28^ This discrepancy is presumed to arise from women's engagement in both professional work and domestic tasks, increasing their workload and associated health problems.

Men had higher mortality rates and a higher probability of death compared to women, with a lower average survival after retirement. This observation may suggest that men retire in poorer health conditions than women.^29^ However, generalizations based on gender can oversimplify behaviors and attitudes, potentially overlooking individual diversity. Furthermore, these characteristics can be influenced by various cultural, social, economic, and educational factors. Therefore, it is crucial to approach such statements cautiously and acknowledge the complexity of the variables involved.

This study found higher mortality rates and shorter survival among professors and those aged 65 to 70 years. Professors are granted the right to dedicate two-thirds of their workload to classroom teaching and the remaining third to scientific production, along with 45 days of vacation per year, unlike other employees. Throughout their careers, this prerogative may result in less physical and mental strain for professors.^30,31^ Thus, they may have a longer active functional life and retire only when affected by more severe illnesses, with a higher probability of death after retirement. Higher mortality at older ages was expected and observed in this study.

The proportion of deaths and mortality rates were higher in the group of full retirements, whether with or without severe cardiovascular disease, compared to the group of proportional retirements. Among the disease groups in full retirements, the neoplasms group had the highest mortality rate, followed by severe heart disease. These findings align with the literature, where cardiovascular diseases remain the leading cause of mortality in both developed and developing countries, showing a trend of reduced incidence and mortality. Meanwhile, neoplasms are increasing globally as a cause of death and already rank as the second leading cause in most countries. In developed countries with aging populations, neoplasms are projected to surpass cardiovascular diseases soon.^32^

In addition to the analyses discussed earlier, it is worth highlighting the findings related to IHD in the context of retirements due to severe heart disease. Among employees retired for this condition, the presence of IHD at the time of retirement emerged as a factor associated with lower mortality rates and higher average survival. This underscores the importance of early detection of IHD in retirement scenarios, potentially positively impacting post-retirement outcomes. When considering the presence of IHD at the time of death, it was observed that deceased employees with this condition had lower mortality rates and higher average survival, although not statistically significant.

Apart from the costs to governments, there are also personal costs for individuals who retire due to IHD. The reduction in income resulting from retirement due to IHD likely decreases individuals’ standard of living and may increase the risk of inadequate retirement savings. A study conducted in Australia reported that workers who retired early due to IHD had significantly less personal wealth and lower savings compared to those who continued working until retirement.^33^ These findings prompt reflection on the necessity of revising current retirement regulations for severe heart disease due to IHD.^17^

Additionally, gender-specific analysis revealed that men had higher mortality rates and lower average survival, indicating gender disparities in risk within the group of retirements due to severe heart disease. These results underscore the complex interactions between heart disease, retirement, and mortality, enhancing understanding of the factors influencing the post-retirement outcomes of these employees.

Finally, in analyzing concordant diagnoses between the illness that led to retirement and the cause of death, in the professor category, there was concordance in more than 70% of cases. This can be explained by the fact that professors retire due to more severe illnesses, thus having a higher probability of death from the same illness. Concordance in the full retirements group also occurred in more than 70% of cases, especially in the group retired due to severe heart disease, indicating greater severity for the illnesses leading to this type of retirement. In contrast, a study in Sweden compared the mortality of 1683 retirees with the non-retired population over 18 years. While a higher mortality rate was found among retirees due to disability, this difference could not be solely attributed to the underlying illness that led to retirement. There was no clear association between the disability-causing diagnosis and the cause of death, suggesting that the underlying illness may not be directly influence the unfavorable outcome.^11^

The absence of a control group consisting of employees who voluntarily retired based on years of service was a limitation of this study for comparing certain variables. Furthermore, the scarcity of similar studies with work contracts governed by the RJU, while contributing to the originality of this study, also limits potential result comparisons.

This study aims to propose hypotheses regarding disability retirements at an early productive age. Its conclusions, though based on data from the largest Federal University in Brazil, can contribute to the development of new studies on the topic. Given recent legislative changes, it is crucial to focus future research on understand the implications of these changes for the living conditions of employees retired due to disability.

## Conclusion

This study provided a clearer understanding of the mortality and survival patterns of employees retired due to disability at UFRJ. The impact of severe heart disease was highlighted, influencing the proportions of deaths and mortality rates, especially in full retirements due to this condition. The significant presence of concordant diagnoses between the cause of retirement and the cause of death emphasizes the vulnerability associated with severe heart disease. The risk of death was higher in full retirements due to severe heart disease. Conversely, survival was longer in proportional retirements and in earlier age ranges, underscoring the importance of periodic post-retirement evaluations to reassess the retirement when the initial disability becomes insubstantial (Central Illustration).

## *Supplemental Materials

For additional information, please click here.
